# The efficacy of pulmonary rehabilitation on cognitive functions in patients with chronic obstructive pulmonary disease: systematic review

**DOI:** 10.3389/fmed.2026.1753968

**Published:** 2026-03-17

**Authors:** Shahad Alkandari, Shibili Nuhmani, Mohammad AlSubaiei, Turki Abualait

**Affiliations:** Department of Physical Therapy, College of Applied Medical Sciences, Imam Abdulrahman Bin Faisal University, Dammam, Saudi Arabia

**Keywords:** cognitive function, cognitive impairment, COPD, pulmonary rehabilitation, rehabilitation

## Abstract

**Background:**

Pulmonary rehabilitation (PR) program is an essential non-pharmacological management in patients with chronic obstructive pulmonary disease (COPD). COPD can lead to cognitive impairment (CI) for about 36%.in COPD patients.

**Objective:**

The aim of this study is to explore the efficacy of PR in improving cognitive functioning in patients with COPD.

**Method:**

Electronic searches of three databases: PubMed, Scopus, and Web of Science were performed. The included studies assessed at least one cognitive function in patients with COPD before and after participating in PR program. Study quality was evaluated by two reviewers independently using risk-of-bias tool (RoB 2) for randomized control trials (RCT) and Risk of Bias In Non-Randomized Studies - of Interventions (ROBIN-I) for non-RCT.

**Result:**

Eleven studies (six RCT and five non-RCT) were selected based on the inclusion criteria, representing ten components of PR intervention, and 25 cognitive tests (or subtests). The total number of participants was 864 patients with COPD. The age range was 48 to 76 years and both genders were included. The studies showed heterogeneity in the study design, PR intervention elements and cognitive tests. PR program showed significant improvement among COPD population in cognitive function in nine studies. The most frequent test used was Montreal Cognitive Assessment test and the most frequent PR component performed was aerobic exercise.

**Conclusion:**

PR programs possibly improve cognitive function among patients with COPD especially those suffering from cognitive impairment. Future studies need to unify the PR intervention elements and the neuropsychological battery tests used to enable a proper evaluation of the program efficacy and application.

**Systematic review registration:**

PROSPERO Registration ID: CRD42023471801.

## 1 Introduction

Cognition refers to the complex neural processes that facilitate the transmission and processing of information influenced by various emotional, psychological, and neurological factors (1). Cognition has also been defined as the intellectual or mental process by which an individual acquires, comprehends, uses, and explores information or knowledge derived from the senses, perception, and thinking, enabling the modification of an individual’s behavior to suit new situations (2, 3). The ability to think critically and creatively, including the capacity to formulate and implement strategies, solve problems, and understand complex ideas, is known as cognitive ability (4). The most important neurophysiological domains of cognition include attention/concentration, learning and memory, language, visuospatial and motor function, executive functions, and social cognition/emotions (2, 5). These cognitive domains provide individuals with different complex and essential functions that can determine one’s intellectual and personal skills (3, 6). Deficits in any of these functional domain’s lead to cognitive impairment (CI), a condition that typically severely impairs a person’s ability to perform a primary or complex function (7).

Chronic obstructive pulmonary disease (COPD) is a heterogeneous obstructive lung disease characterized by respiratory symptoms such as dyspnea, sputum production, cough, and exacerbated lung function due to abnormalities of the airways or the alveoli (8). Around 455 million people worldwide were diagnosed with COPD in 2019. COPD is considered a life-threatening condition with a high mortality rate, as it is the third leading cause of death globally, with about 3.2 million deaths (9). In addition to suffering the respiratory symptoms associated with COPD, patients with this condition are prone to various comorbidities, including lung cancer, cardiac disease, anxiety and depression, musculoskeletal problems, and CI (6, 10, 11). According to evidence, about 98% of COPD patients have one or more comorbidities (12). CI is one of COPD’s most prevalent comorbid disorders (6). The CI morbidity in patients with COPD is higher than that of healthy populations with similar characteristics, and about 36% of patients with COPD experience CI compared to 16.7% of the general population (13). COPD patients suffering from CI exhibited impaired disease management and functional ability, which resulted in them having high mortality rates and socioeconomic burdens (14). A meta-analysis of 14 studies concluded that around 32% of individuals with COPD suffer from CI and that about one in four experience mild cognitive impairment (MCI) (15).

The relationship between COPD and CI has been hypothesized several times. COPD has several risk factors, with a primary factor being smoking, which has been known to increase the likelihood of developing CI (16). The various toxic components of tobacco, such as carbon monoxide and nicotine, can damage nerve cells and impair their function (17). Moreover, individuals with COPD frequently experience chronic sleep deprivation, which is associated with impaired cognitive function, as sleep is essential for mental processes, and insufficient sleep can disrupt brain clearance mechanisms and reduce blood flow in the prefrontal cortex, which may contribute to neuronal death in regions responsible for sleep-related functions (18–20). Therefore, sleeping disturbance comorbidities in COPD might lead to CI. According to previous studies, COPD can trigger the development of CI due to various mechanisms, such as tissue hypoxemia, systemic inflammation, hypercapnia, and oxidative stress (21–23). The reduction of oxygen availability due to lung dysfunction is the most independent risk factor that can lead to CI in patients with COPD (6). From a physiological perspective, the brain is reasonably assumed to have the highest oxygen demand in the body; therefore, it is highly susceptible to ischemia (3). When the oxygen supplied to the brain is insufficient to meet its needs, neurons that synthesize neurotransmitters, such as acetylcholine, are lost, leading to brain atrophy and cognitive deficits, including white matter degeneration (13, 24, 25). This sequence will eventually lead to CI. Moreover, patients who experience consistent cerebral hypoxia are prone to experiencing various cognitive decline-related symptoms, such as declines in attention, information processing, memory, visuospatial abilities, judgment, language, and executive functioning (3, 25, 26). Patients afflicted by CI may face difficulty managing their conditions and keeping track of their symptoms or medications (25, 27). Therefore, patients with COPD who also suffer from CI have worsened their condition over time.

Pulmonary rehabilitation (PR) is a vital, comprehensive, non-pharmacological, and evidence-based intervention for COPD patients that includes several components, such as exercise, education, and nutrition (28–31). Numerous systematic reviews have shown that PR can improve exercise capacity, quality of life, and self-management, particularly in controlling disease and emotional functioning among patients with COPD (32–35). A recent review reported that PR is a promising management strategy for improving the health-related quality of life (HRQoL) of COPD patients through programs involving education, exercise training, and breathing exercises (36). PR for patients with COPD has been proven to exert psychological benefits and reduce healthcare resources, and it has been shown to facilitate low-cost management (37). Patients suffering from COPD who had experienced an exacerbation were likely to have lower mortality rates, hospitalization rates, and readmission rates following their initial PR sessions (38).

Despite all previously offered evidence on the effectiveness of the PR program in improving patients’ condition across several aspects, evidence on PR’s efficacy in improving cognitive function in patients with COPD and cognitive impairments is lacking. The primary focus of the outcome measures in previous studies was the effectiveness of PR on exercise capacity and HRQoL; however, no specific outcomes have been used to evaluate cognitive function in patients with COPD practically (32–35). A limited number of studies have examined the effects of PR interventions on cognition in individuals with COPD (39–42). In addition, these studies did not provide conclusive evidence on the effects of PR interventions on cognitive function in individuals with COPD. Andrianopoulos et al. reported that patients with COPD, with or without CI, showed significant improvement in cognitive function post-PR (41). In contrast, other studies found that patients with COPD and CI showed significant improvement in cognitive function, whereas those without CI did not (40, 42). Furthermore, Bonnevie et al. found that the score of the cognitive test was not significantly associated with the score of other outcomes such as functional capacity, quality of life (QoL), and anxiety and depression scale, although their results obtained through the cognitive test remain questionable due to the absence of minimal clinical important difference value for the Montreal Cognitive Assessment tool (MoCA) (42). Moreover, Tabka et al. concluded that PR significantly improves cognitive function in COPD patients with CI, and that incorporating cognitive-behavioral therapy into PR resulted in greater improvements than PR alone (39). Therefore, no clear conclusion regarding the effects of PR interventions on the cognitive function of individuals with COPD currently exists.

Earlier reviews have looked at how physical activity or exercise-based interventions affect cognitive function in people with COPD. For example, Desveaux et al. (43) studied the cognitive benefits of exercise and lifestyle physical activity. Still, these reviews did not focus on structured pulmonary rehabilitation programs or clearly separate different cognitive domains. However, this review aims to fill those gaps by evaluating pulmonary rehabilitation, examining cognitive outcomes by specific domains, and taking baseline cognitive status into account. This approach offers a more clinically useful and detailed summary of the current evidence.” To date, no systematic review has examined the effects of PR interventions on cognitive function in individuals with COPD. For this reason, this systematic review aims to explore articles on the effectiveness of PR in improving cognitive functioning in patients with COPD. We anticipate that this review’s results will serve as a valuable guide in the decision-making process for enrolling patients with COPD who suffer from CI in PR programs, and we consider this intervention an essential component in managing this population.

The following research question is addressed in this review to test the efficacy of PR on the cognitive function of patients with COPD: How effective is PR at improving the cognitive function of patients with COPD?

P: Patients with COPD.
I: PR program.
C: None.
O: Cognitive function.

## 2 Methods

This systematic review adhered to the Preferred Reporting Items for Systematic Reviews and Meta-Analysis 2020 (PRISMA 2020) guidelines (44). PRISMA checklist has been added as Supplementary material 1. The study protocol has been registered in PROSPERO with the following ID: CRD42023471801.

### 2.1 Search strategy

PubMed, Scopus, and Web of Science databases were searched for published articles. Searching was conducted from January 14, 2024, to January 24, 2024. The following Medical Subject Headings (MeSH) search terms used were as follows: (“cognition” OR “cognition disorders” OR “cognitive dysfunction” OR “attention” OR “memory”) AND (“neuropsychological tests” OR “neuropsychological battery test” OR “neuropsychological assessment” OR “cognitive test” OR “cognitive assessment”) AND (“pulmonary rehabilitations” OR “respiratory rehabilitation” OR “exercise” OR “physical therapy”) AND (“chronic respiratory disease” OR “chronic obstructive pulmonary disease” OR “chronic bronchitis” OR “emphysema”). Details for the search strategy employed for each database are available on Supplementary material 2.

The results were limited to published English-language articles that involved humans and were full-text, peer-reviewed.

### 2.2 Process

Two independent reviewers (MA and SA) screened the resulting articles. The same reviewers checked the reference lists of each full-text article to determine whether any additional articles should be included. Moreover, disputes and conflicts were resolved by having the reviewers reach an agreement.

### 2.3 Study eligibility

The search results were limited to published English-language articles that involved humans and were full-text, peer-reviewed. Studies on randomized controlled trials (RCTs), non-randomized controlled trials (non-RCTs), prospective observational and cohort designs conducted on COPD patients who underwent PR programs, and the cognitive functions assessed, were included in our study. These articles must consist of at least one cognitive outcome evaluated with neuropsychological tests that are sufficiently sensitive to detect alterations in cognitive function and to provide further information across cognitive domains. Studies that explored patients with other respiratory diseases, did not test cognitive function, or used interventions other than PR were excluded. In addition, case reports, case series, review articles, qualitative investigations, papers that included book chapters, protocol papers, reviews, conference abstracts, and articles that did not fully represent the selected outcomes were also excluded.

### 2.4 Quality assessment for risk of bias

The included studies were assessed using either Risk of Bias in Randomized Trials (RoB 2) (45) for RCTs or risk of bias in non-randomized studies of interventions (ROBINS-I) (45) for non-RCTs (46), for non-RCTs. RoB 2 was used with respect to its five domains: (1) bias arising from the randomization process, (2) bias due to deviations from intended interventions, (3) bias due to missing outcome data, (4) bias in measurement of the outcome, and (5) bias in selection of the reported result. The ROBINS-I tool was used with respect to its seven domains: (1) confounding; (2) selection of participants; (3) classification of interventions; (4) deviation from intervention; (5) missing data; (6) measurement of outcome; (7) selection of reported result. Two reviewers (MS and SA) used these tools independently. Conflicts and disputes were resolved upon agreement between the reviewers.

### 2.5 Data extraction

The data extraction process included: sample size, age, gender, component of the PR program, program duration, and cognitive function outcome measures. PR was defined as a structured intervention including at least one core component of PR (e.g., exercise training, respiratory muscle training, education, or multidisciplinary management), delivered with the aim of improving functional and health-related outcomes (30). These data are essential for determining the differences between the programs, the subjects’ characteristics, and the measured outcomes. Two reviewers (MA and SA) extracted these data and inserted them into IBM SPSS Statistics, Version 21 (IBM Corp., Armonk, NY, USA) for statistical analysis.

## 3 Ethical statement

Ethical approval was not required for this study because it is a systematic review of previously published studies and did not involve the collection of new data from human participants.

## 4 Results

### 4.1 Search results

**Figure 1:**
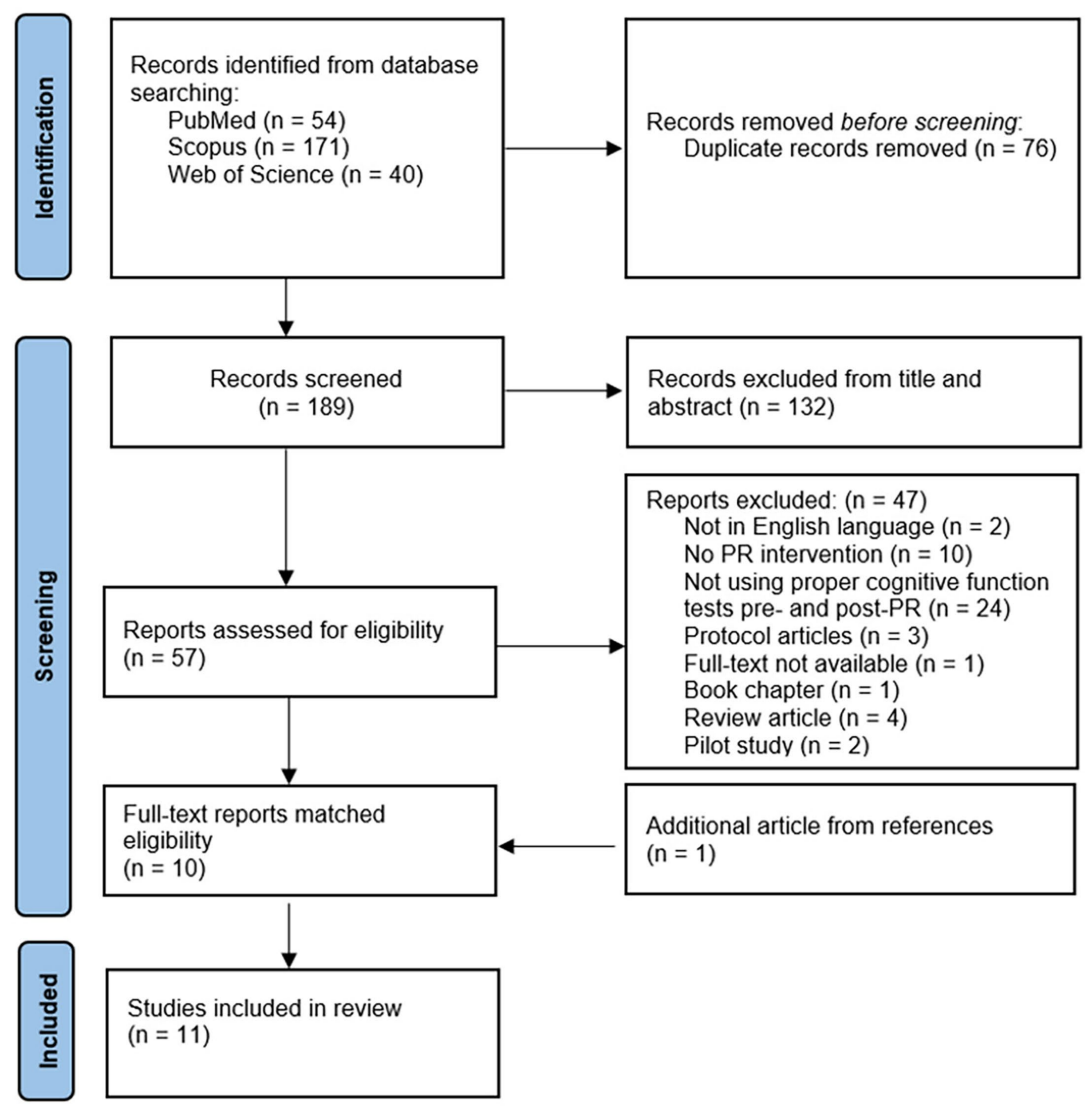
PRISMA flow diagram.

The comprehensive search across multiple databases yielded an initial identification of 256 articles. Following the removal of 76 duplicate records, the titles and abstracts of 186 articles were screened, and 57 articles were retrieved for full-text analysis based on eligibility criteria. Ten of the 57 articles met the eligibility criteria, and one additional study was added from the references of the selected studies after a manual search. The selection process of the included studies is outlined in Figure 1. The characteristics of the studies and their results are shown in Tables 1, 2, respectively. A total of 11 studies were included in this systematic review, representing 10 different components of PR intervention. Furthermore, 25 different cognitive tests (or subtests) were used among the studies. A total of 864 patients with COPD participated in the studies included in this review. These participants ranged in age from 48 to 76.1 years.

**Table 1:** Summary of included studies in the systematic review.

References	Design	Participants^a^	Intervention	Control	Cognitive assessment
Emery et al., 1998 (52)	RCT	N_total_ = 79 *Waitlist control*: *n* = 25, age: 67 ± 6 yrs.^a^, 48% male *Exercise intervention*: *N* = 29, age: 65 ± 7 yrs.^a^, 50% male	*Duration*: 10 weeks *Intensity*: Not specified *Frequency*: 3–5 days/week *Components*: First 5 weeks (5 days/week): 45 min of aerobic exercises and strength training. 60 min of education (4 days/week) 60 min of stress management (1 time/week) Last 5 weeks (3 days/week): 60–90 min of exercises. 60 min of stress management (1 day/week).	*Waitlist control*: No intervention *Educational control*: Educational lectures and stress management sessions only	Digit Vigilance Test, Verbal Fluency Test, Finger Tapping Test, Trail Making Test Digit Symbol subscale of WAIS-R.
Kozora et al., 2002 (54)	Experimental, Non-RCT	N_total_ = 59 *Control*: *n* = 29, age: 67 yrs.^b^, 45% male *Exercise intervention*: *n* = 30, age: 67 yrs.^b^, 50% male	*Duration*: 3 weeks *Intensity*: Not specified *Frequency*: 4 days/week *Components*: Exercise, educational and psychosocial sessions.	COPD control with no intervention	Digit Span and Digit Symbol subscales of WAIS-R, Logical Memory, Visual Reproduction, and Paired Associates subscales of WMS-R, Trail Making Test, Digit Vigilance Test, Clock Drawing to Command, Boston Naming Test, Animal Naming Test Controlled Oral Word Association Test
Pereira et al., 2011 (53)	Cohort: Prospective study	N_total_ = 34, age: 65 ± 7 yrs.^a^, 50% male	*Duration*: 3 months *Intensity*: Not specified *Frequency*: 3 days/week *Components*: Resistance, aerobic training, psychosocial and educational sessions	None	Stroop Test, Digit Span Test, FAS Test, RAVLT
Aquino et al., 2016 (47)	RCT	N_total_ = 28 *Resistance and aerobic exercise*: *n* = 14, age: 65 ± 8 yrs.^a^, 100% male *Aerobic exercise alone*: *n* = 14, age: 69 ± 7 yrs.^a^, 100% male	*Duration*: 4 weeks *Intensity*: progressing over 4 weeks *Resistance training session*: 4–10 rep in 3 sets at 70–90% of 1-RM *Aerobic training session*: 30 min at 70–90% of HRmax *Frequency*: 2 times/day for 5 days/week *Components*: Resistance training, aerobic training, respiratory, mobility and balance exercises.	Aerobic Exercise alone	RAVLT, Attentive Matrices Test, Drawing Copy Test, Verbal Fluency Test, Raven Test
Bonnevie et al., 2020 (42)	Prospective multicentre observational study	N_total_ = 56 Group with MoCA < 26: *n* = 41, age: 64 ± 9 yrs.^a^, 51% male Group with MoCA ≥ 26: *n* = 15, age: 59 ± 11 yrs.^a^, 33% male	*Duration*: 8 weeks *Intensity*: *Peripheral muscle strengthening*: 70% of the 1-RM *Endurance training*: adjusted at the anaerobic threshold then increased based on symptoms. *Frequency*: 3 days/week *Components*: Respiratory physiotherapy, endurance training, muscle strengthening, nutritional support, and self-management.	None	MoCA
Incalzi et al., 2008 (51)	RCT	N_total_ = 105 *Control group*: *n* = 52, age:68.3 ± 7.8 yrs.^a^, 84.6% male *CogT training group*: *n* = 53, age: ± 8.2 yrs.^a^, 86.8% male	*Standardized program*: Standardization of pharmacological therapy, inhalers according to patient’s ability, nutritional counseling, respiratory rehabilitation, oxygen therapy, health education, control visits *For respiratory rehabilitation*: Inpatient program *Duration*: 6 weeks *Intensity*: up to 70% of the Wmax *Frequency*: Not specified *Components*: upper arm exercises and inspiratory muscle training. Home program *Duration*: 6 months from baseline *Intensity*: Not specified *Frequency*: 30 min daily *Components*: Not specified CogTprogram: Inpatient program *Duration*: 6 weeks *Frequency*: 15 min each training *Components*: 4 cognitive function training Home program *Duration*: 6 months from baseline *Frequency*: 2 days/week, booster sessions at 3 and 5 months *Components*: Same as inpatient program in addition booster session	Usual care (Standardized program only)	Raven’s progressive matrices test, Verbal fluency test, Visual span test, Verbal span test, Verbal memory—short term test, Verbal memory—long term test, Copying drawings test, Copying drawings with landmarks test, Immediate visual memory test, Sentence construction test, Number of defective MDB tasks, MMSE score
Lavoie et al., 2019 (50)	Randomized, partially double-blind, placebo-controlled, parallel-group trial	N_total_ = 304 SMBM+T group: *n* = 76, age; 65.1 ± 6.4 yrs.^a^, 72.4% male SMBM+T/O group: *n* = 76, age: 64.9 ± 6.9 yrs.^a^, 63.2% male SMBM+T/O + ExT group: *n* = 76, age: 64.8 ± 6.5 yrs.^a^, 59.2% male SMBM+P group: *n* = 76, age: 64.4 ± 6.6 yrs.^a^, 69.7% male	SMBM program: *Duration*: 12 weeks *Frequency*: 1 time/day *Components*: Behavior change techniques (e.g., goal setting, positive reinforcement and problem solving) ExT program: *Duration*: 8 weeks *Intensity*: Gradually increased based on dyspnoea and fatigue score. *Frequency*: 3 times/week *Components*: Hight-intensity of whole-body exercises training, and lower limb resistance training.	SMBM+P group (SMBM with placebo)	MoCA
France et al., 2021 (40)	Prospective Observational study	N_total_ = 112 *AECOPD group*: *n* = 45, age: 68 (61.5–74.0) yrs.^c^, 60% male *PR group*: *n* = 67, age: 69 (64.0–73.0) yrs. ^c^, 55.2% male	*Duration*: 6 weeks *Intensity*: Not specified *Frequency*: 2 h/day, 2 days/week *Components*: Resistance and aerobic training, multidisciplinary disease-related education	AECOPD group: (no intervention; natural recovery)	MoCA
Cheng et al., 2022 (48)	Prospective study	N_total_ = 48 Total age: 67.23 ± 7.32 yrs.^a^, 100% male *IMT + EMT group*: *n* = 28 *IMT group*: *n* = 20	*Duration*: 8 weeks *Intensity*: Not specified *Frequency*: 2 times/day, 5 days/week *Components*: 30 breaths for IMT and EMT	IMT group: (only IMT)	MMSE
Kaya et al., 2023 (49)	RCT	N_total_ = 24 *PT+CDE Group*: *n* = 12, age: 65.17 ± 6.35 yrs.^a^, 83.3% male *PT Group*: *n* = 12, 64.75 ± 8.49 yrs.^a^, 83.3% male	PT program: *Duration*: 8 weeks *Intensity*: For physical activities: Moderate-intensity aerobic *Frequency*: For beathing exercise:2 sets with 5 rep 2 times/days, 5 days/week *For physical activities*: 30 min 5 days/week *Components*: For breathing exercises: Diaphragmatic, chest, and bilateral segmental breathing exercises, incentive spirometer, the teaching of breathing control, relaxation of body positions, coughing techniques, and pursed lip breathing. *For physical activities*: walking, cycling, and swimming. The CDE: *Duration*: 8 weeks *Intensity*: Not specified *Frequency*: 45 min. *Components*: Locomotor and non-locomotor movements combined with elements of dance	PT group: (receiving the standard PT program)	MoCA
Tabka et al., 2023 (39)	RCT	N_total_ = 39 *PR + CogT group*: *n* = 21, age: 65.2 ± 2.79 yrs.^a^, 100% male *PR group*: *n* = 18, age of 65.3 ± 3.2 yrs.^a^, 100% male	PR program *Duration*: 12 weeks *Intensity*: 60–70% of MHR *Frequency*: 40 min exercise session, 3 days/week *Components*: Endurance training, education for COPD management, symptom monitoring, breathing techniques and medical management. CogT program *Duration*: 12 weeks *Intensity*: The difficulty increased progressively *Frequency*: 20 min each session 3 days/week *Components*: Paper-and-pencil exercises: attention exercise, memory exercise, concentration exercise, language exercise.	PT group: (receiving the standard PT program)	P 300 MoCA

RCT, randomized controlled trial; N, number of participants in the study; *n*, number of participants in the group; Yrs, years; WAIS-R, Wechsler Adult Intelligence Scale – Revised; WMS-R, Wechsler Memory Scale – Revised; RAVLT, Rey Auditory Verbal Learning Test; FAS, oral fluency test by letters F, A, and S; 1-RM, One Repetition Maximum; HR, heart rate; MoCA, Montreal Cognitive Assessment test; W, watt; CogT, cognitive training; MMSE, the Mini Mental State Examination; SMBM, Self-Management Behavior Modification; T, Tiotropium 5 μg; T/O, Tiotropium/Olodaterol 5/5 μg; ExT, Exercise Training; P, placebo; AECOPD, Acute Exacerbation of Chronic Obstructive Pulmonary Disease; PR, Pulmonary Rehabilitation; IMT, Inspiratory Muscle Training; EMT, Expiratory Muscle Training; PT, Physiotherapy; CDE, Creative Dance-based Exercise; MIN, Minutes.

^a^Age reported as mean ± standard deviation.

^b^Standard deviation not provided.

^c^Age reported as median (interquartile range).

**Table 2:** Significance of pre-to-post within subject in cognitive measures across studies.

Study design	RCT						Non-RCT				
	Emery et al., 1998 (52)	Aquino et al., 2016 (47)	Incalzi et al., 2008 (51)	Lavoie et al., 2019 (50)	Kaya et al., 2023 (49)	Tabka et al., 2023 (39)	Kozora et al., 2002 (54)	Pereira et al., 2011 (53)	Bonnevie et al., 2020 (42)	France et al., 2021 (40)	Cheng et al., 2022 (48)
Cognitive domains and tests											
Processing speed/attention/executive functions											
Finger Tapping	NS	-	-	-	-	-	-	-	-	--	-
Digit Vigilance	NS	-	-	-	-	-	IS only: 0.004	-	-	-	-
Digit Span (Visual span)	-	-	NS	-	-	-	NS	NS	-	-	-
Digit Symbol	NS	-	-	-	-	-	NS	-	-	-	-
Stroop Colour Word Test 1	-	-	-	-	-	-	-	0.024	-	-	-
Trail Making Test	-										
Part A	NS	-	-	-	-	-	-	-	-	-	-
Part B	NS						NS				
Verbal Fluency Test (Letter) (FAS)	<0.001	-	NS	-	-	-	NS	NS	-	-	-
Verbal Fluency Test (Semantic)	-	CT: <0.01 AT: <0.01 Both: <0.01	-	-	-	-	NS	-	-	-	-
Verbal Span	-	-	NS	-	-	-	-	-	-	-	-
Verbal Competence (Sentence Construction)	-	-	NS	-	-	-	-	-	-	-	-
Attentive Matrices Test	-	CT: <0.01 AT: <0.01 Both: <0.01	-	-	-	-	-	-	-	-	-
Learning and Memory											
RAVLT	-		-	-	-	-	-	-	-	-	-
Sum of Trials 1 to 5	-	-	-	-	-	-	-	<0.001	-	-	-
Short Delay Recall (REY-IR)	-	NS	NS	-	-	-	-	0.005	-	-	-
Long Delay Recall (REY-DR)	-	CT: <0.01 AT: <0.05 CT vs. AT: <0.05	NS	-	-	-	-	0.022	-	-	-
Logical Memory (Story Retention)	-		-	-	-	-	IS only: 0.006	-	-	-	-
Visual Reproduction (Visual Retention)	-		-	-	-	-	NS	-	-	-	-
Paired Associates (Verbal Pairs)	-		-	-	-	-	NS	-	-	-	-
Immediate Visual Memory	-		NS	-	-	-	-	-	-	-	-
Visuo-spatial abilities											
Clock Drawing	-		-	-	-	-	NS	-	-	-	-
Drawing Copy Test I	-	NS	NS	-	-	-	-	-	-	-	-
Drawing Copy Test II	-	CT: <0.01 AT: <0.05 CT vs. AT: <−0.05	NS	-	-	-	-	-	-	-	-
Language											
Boston Naming Test	-	-	-	-	-	-	NS	-	-	-	-
Intelligence and abstract reasoning											
Raven Test	-	CT: <0.01 AT: <0.05 CT vs. AT: <0.05	NS	-	-	-	-	-	-	-	-
Multiple cognitive domains											
MMSE (Total score)	-	-	NS	-	-	-	-	-	-	-	IMT: NS IMT + EMT: NS Total: 0.002
MoCA (Total score)	-	-		SMBM+T: <0.001 SMBM+T/O: <0.001 SMBM+T/O + ExT: <0.001 SMBM+P: <0.001 SMBM+T/O vs. SMBM+P: <0.05 SMBM+T vs. SMBM+P: <0.05	PT + CDE: < 0.05 PT: < 0.05 PT + CDE vs. PT: <0.05	IG: <0.001 CG: <0.001 IG vs. CG: <0.001	-	-	MoCA<26: <0.01 MoCA>26: NS Total: <0.01	IS only: 0.004	-
MoCA (Sub-category score)											
Visuospatial	-	-		NA	NA	NA	-	-	NS	NA	-
Naming	-	-		NA	NA	NA	-	-	NS	NA	-
Attention	-	-		NA	NA	NA	-	-	NS	NA	-
Language	-	-		NA	NA	NA	-	-	NS	NA	-
Abstract	-	-		NA	NA	NA	-	-	NS	NA	-
Memory	-	-		NA	NA	NA	-	-	MoCA<26: <0.01	NA	-
Orientation	-	-		NA	NA	NA	-	-	NS	NA	-

RCT, randomized controlled trial; NS, not significant; NA, not available; FAS, oral fluency by letters F, A, and S; RAVLT, Rey Auditory Verbal Learning Test; MoCA, Montreal Cognitive Assessment test; MMSE, the Mini Mental State Examination; SMBM, Self-Management Behavior Modification; T, Tiotropium 5 μg; T/O, Tiotropium/Olodaterol 5/5 μg; ExT, exercise training; P, placebo; IMT, Inspiratory Muscle Training; EMT, expiratory muscle training; PT, physiotherapy; CDE, creative dance-based exercise; CT, combined aerobic and resistance training; AT, aerobic training only; IG, intervention group; CG, control group; IS only, impaired subject only.

Both genders were included, except in three studies in which only male participants were included (39, 47, 48).

### 4.2 Risk of bias across outcomes

#### 4.2.1 Risk of bias across RCT using the RoB 2 tool

**Figure 2:**
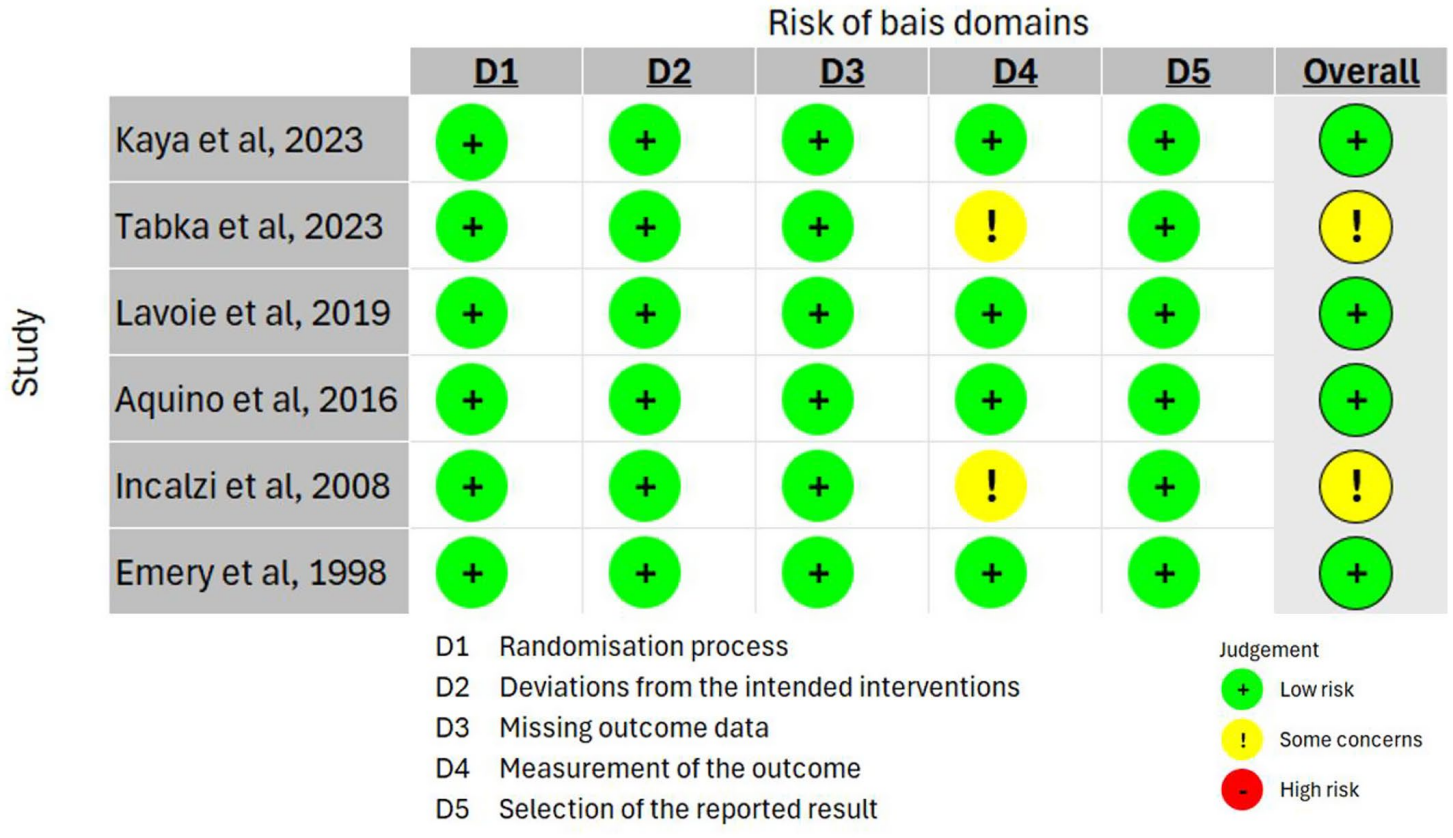
Risk of bias assessment of the selected studies using RoB2 tool.

The included RCTs’ risk of bias was comprehensively evaluated using the RoB 2 tool, which assesses biases across five domains. In line with the research plan to examine the effects of PR on cognitive function among COPD patients, the studies were evaluated using the intention-to-treat (ITT) analysis method. A summary of the review’s findings on the studies’ risk of bias is presented in Figure 2.

##### 4.2.1.1 Randomization process

The reduction of selection bias in clinical trials is primarily dependent on the trials’ randomization processes. In this review, all RCTs indicated a low risk of bias in the randomization processes. Kaya et al. and Tabka et al. used a computer-based program (39, 49) for randomization, while Lavoie et al. used a pseudorandom number generator (50). In addition, three studies used simple randomization (47, 51, 52). Moreover, Tabka et al. and Emery et al. clearly stated that the allocation sequence was concealed (39, 52). This strict approach implies high confidence in allocating individuals to the control or intervention groups. Furthermore, Lavoie et al. and Aquino et al. used a block-randomized design to achieve balanced allocation (47, 50).

##### 4.2.1.2 Deviations from intended interventions

This section evaluates adherence to and consistency of the intervention protocols. In this domain, all included RCTs demonstrated low risk of bias, indicating that the interventions were delivered as planned (39, 47, 49–52).

##### 4.2.1.3 Missing outcome data

Comprehensive outcome data are essential for the integrity of the study’s findings. Every RCT study in our review was rated as low risk, suggesting that adequate data were available for evaluation and analysis (39, 47, 49–52).

##### 4.2.1.4 Measurement of outcomes

Outcomes must be measured reliably and consistently to validate results. Most RCTs in our review had a low-risk score in this domain, which makes the reported findings more credible (47, 49, 50, 52). However, the works of Tabka et al. and Incalzi et al. raised concerns in this domain, as the outcome assessors were not blinded to or unaware of the intervention the research participants had received (39, 51).

##### 4.2.1.5 Selection of the reported result

The trustworthiness of the study is impacted by the transparency with which the results are presented. All RCTs included in our review had low risk of bias in reporting, indicating high accountability and transparency (39, 47, 49–52).

##### 4.2.1.6 Overall bias

Most included studies were judged to have a low risk of bias. However, some concerns were seen only in one domain: outcome measurement. Moreover, none of the studies included were categorized as having a high risk of bias, indicating their overall reliability and quality.

#### 4.2.2 Risk of bias across non-RCT studies using the ROBIN-I tool

**Figure 3:**
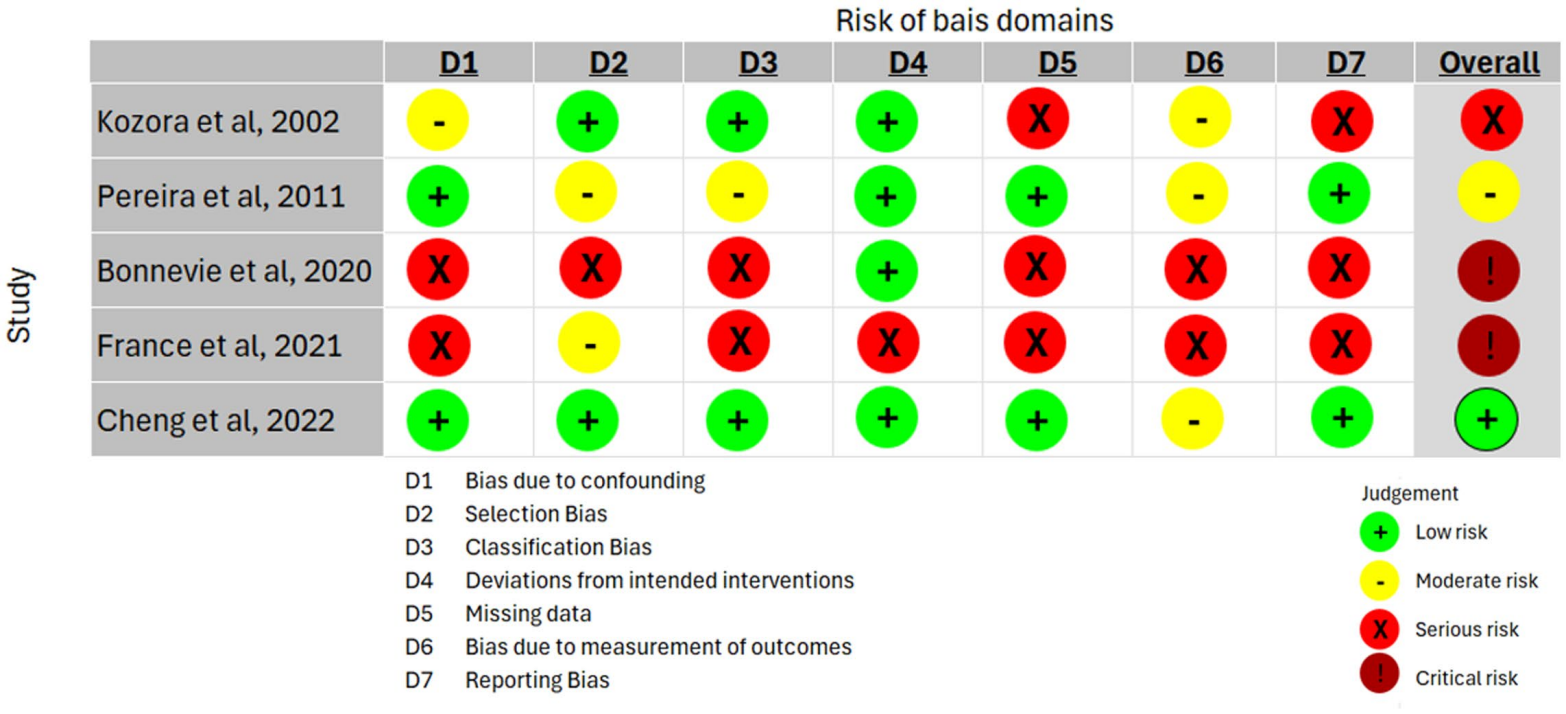
Risk of bias assessment of the selected studies using ROBBIN-I tool.

The risk of bias of the included non-RCT studies was comprehensively evaluated using the ROBIN-I tool, which assesses biases across seven domains. In line with the research plan to examine the effects of PR on cognitive function among COPD patients, the included studies were evaluated using the ITT analysis method. A summary of the findings on risk of bias is presented in Figure 3.

##### 4.2.2.1 Bias due to confounding (pre-intervention)

Determining a specific causal relationship between interventions and outcomes might be complicated by confounding variables. Thus, the use of proper techniques to account for confounding variables is crucial. The studies by Pereira et al. and Cheng et al. showed low risk of bias in this domain, as there were no significant differences in demographic characteristics between the groups (48, 53). While Kozora et al.’s study was rated as having a moderate risk of bias due to a significant baseline difference between the groups, the results remained significant after adjusting for baseline differences (54). Moreover, the work of Bonnevie et al. and France et al. indicated a serious risk of bias in this domain due to dividing the groups into a CI group and a non-CI group, where CI is considered one of the main confounding factors that will affect the response to the treatment applied and, therefore, the results (40, 42).

##### 4.2.2.2 Bias in the selection of participants into the study (pre-intervention)

Bias in selection may arise from the initial follow-up period of specific individuals or the exclusion of eligible participants. Even if the intervention’s effects are the same, there is still a correlation between the intervention and the outcome. A low risk of bias score in this domain was found in the studies of Cheng et al. and Kozora et al. (48, 54). In contrast, Pereira et al. and France et al. demonstrated a moderate score risk of bias in this domain: Pereira et al. confirmed a significant difference in neuropsychological testing between groups at the baseline, and France et al. also showed a significant difference in the orientation domain of the cognitive test between the groups at the baseline (40, 53). Furthermore, Bonnevie et al. rated the risk of bias as serious due to the separation of the groups according to their CI, as this would imply an association between the intervention and outcomes (42).

##### 4.2.2.3 Bias in the classification of interventions (during intervention)

The bias arises from incorrectly classifying intervention status as either differentially or non-differentially. Kozora et al. and Cheng et al. scored low in this section, while Pereira et al. exhibited a moderate score (48, 53, 54). The work of Pereira et al. indicated differences in the baseline cognitive assessment between groups, and only the experimental group was enrolled in PR; thus, there was bias in the intervention (53). Furthermore, Bonnevie et al. and France et al. reported serious risk of bias due to the division of the group into CI and non-CI, which influenced the intervention because CI is considered a cofounding factor, and due to the subcategories of CI and non-CI participants, respectively (40, 42).

##### 4.2.2.4 Bias due to deviations from intended interventions (post-intervention)

This domain reflects a deviation from the intended treatment. The effect of interest determines how bias is assessed in this area. All non-RCT studies demonstrated a low risk of bias in this domain (42, 48, 53, 54) except France et al., who achieved a moderate score due to the effect of starting and adhering to the intervention (40).

##### 4.2.2.5 Bias due to missing data (post-intervention)

Bias resulting from the absence of subsequent follow-up for individuals initially included and monitored. Pereira et al. and Cheng et al. demonstrated a low risk of bias in this domain (48, 53); however, the other non-RCTs studies, which were conducted by Kozora et al., Bonnevie et al., and France et al., showed serious scores due to the large number of participants who lost follow-up (40, 42, 54).

##### 4.2.2.6 Bias in measurement of outcomes (post-intervention)

Bias resulting from measurement inaccuracies in outcome data, whether the data are differential or nondifferential. In this domain, Kozora et al., Pereira et al., and Cheng et al. reported moderate scores due to their inability to blind the assessor (48, 53, 54). Meanwhile, Bonnevie et al. and France et al. indicated a serious risk of bias due to the presence of CI and non-CI categories between the groups in their studies (40, 42).

##### 4.2.2.7 Bias in the selection of the reported result (post-intervention)

Results are reported selectively based on the findings. Pereira et al. and Cheng et al. scored low risk of bias in this domain, as they reported their data correctly (48, 53). However, Bonnevie et al. and France et al. indicated a serious risk of bias due to the inappropriate sequence of group selection and the presence of a confounding factor: CI (40, 42). In addition, Kozora et al. also showed a serious risk of bias because, in some paragraphs, they stated that no significant interaction effects were found. In contrast, in other paragraphs, they reported significant interaction effects for the same tests (54).

##### 4.2.2.8 Overall bias

Only Cheng et al. reported a low-risk score for overall bias (48). Only one study, conducted by Pereira et al., showed a moderate score (53), and only one study, Kozora et al., showed a serious score (54). Both Bonnevie et al. and France et al. demonstrated a critical risk of bias (40, 42). Although the overall results were not low in most included studies, they were still included in this review because the limited matching study designs were used for the review’s purpose.

### 4.3 Intervention component delivery

The components of the intervention varied, including aerobic exercise in 10 studies (39, 40, 42, 47, 49–54), strengthening exercises in six studies (40, 42, 47, 50, 52, 53), education sessions in six studies (39, 40, 47, 51, 52, 54), breathing exercises in four studies (39, 42, 49, 53), medical management in three studies (39, 50, 51), and nutritional sessions in two studies (42, 51), stress management in two studies (52, 54), respiratory muscle training in two studies (48, 51), psychological session in one study (53), and balance exercises in one study (47). A description of the intervention components is provided in Table 1. Eight studies examined the PR intervention as one or more components within the subjects (40, 42, 47, 48, 50, 52–54). In contrast, other studies included PR with other interventions; two studies included PR and cognitive training (CogT) (39, 51), and one study included PR and creative dance-based exercise training (CDE) (49).

#### 4.3.1 Effect of PR versus control group with no intervention on cognitive function

Three studies evaluated the effects of PR versus a control group without intervention (40, 52, 54). The RCT by Emery et al. (52) showed significant improvement (*p* < 0.001) in the letter-verbal fluency task in favor of the intervention group; the remaining tests were not significant. One non-RCT study found no significant between-group differences except for cognitively impaired subjects in digit vigilance (*p* = 0.004) and logical memory (story retention) (*p* = 0.006) in favor of PR (54). The other non-RCT study by France et al. (40) reported a significant improvement in favor of the PR group (*p* = 0.004) only among cognitively impaired subjects.

#### 4.3.2 Effect of PR versus control group with other PR components on cognitive function

Four studies examined the effects of PR versus the control group with other PR components (47, 48, 50, 52). The first study (47) compared the combination of aerobic and strengthening training (CT) versus aerobic training (AT) alone. It revealed that both groups improved significantly in almost all cognitive tests: Verbal Fluency Test (Semantic) (CT: *p* < 0.01, AT: *p* < 0.01), Attentive Matrices Test (CT: *p* < 0.01, AT: *p* < 0.01), Long Delay Recall (REY-DR) (CT: *p* < 0.01, AT: *p* < 0.05), Drawing Copy Test II (CT: *p* < 0.01, AT: *p* < 0.05), and Raven Test (CT: *p* < 0.01, AT: *p* < 0.05). Additionally, CT was significantly superior to AT (CT vs. AT: *p* < 0.05) in the three following tests: REY-DR, Drawing Copy Test II, and Raven Test. However, two tests, the Short Delay Recall (REY-IR) and the Drawing Copy Test I, showed no significant improvement in either group. The second study compared the intervention group (IG) with aerobic strengthening, education, and self-management to the control group (CG) with education and self-management alone and showed no statistically significant difference in all cognitive tests except in the Verbal Fluency Test (Letter) (FAS) in favor of IG (*p* < 0.001) (52). The third study (50) conducted an RCT for four groups: Self-Management Behavior Modification (SMBM) and Tiotropium 5 μg (SMBM+T), SMBM with Tiotropium/Olodaterol 5/5 μg (SMBM+T/O), SMBM with T/O and exercise training (SBMB+T/O+ExT), and SMDM with placebo (SMBM+P) and examined the cognitive function by MoCA. Total MoCA scores improved significantly across all groups (*p* < 0.001). Additionally, SMBM+T/O and SMBM+T were superior to SMBM+P (*p* < 0.05) (50). The fourth study compared the combination of inspiratory muscle training (IMT) and expiratory muscle training with IMT alone and revealed no significant differences among groups in terms of the Mini-Mental State Examination (MMSE) score; however, combining both group results indicated significant improvements in MMSE scores with *p*-value (*p* < 0.002) (48).

#### 4.3.3 The effect of PR as a standard program versus other interventions on cognitive function

Two RCTs compared the standard program as the CG and the integration of the standard program with CT as the IG (39, 51); however, both studies reported conflicting results. Incalzi et al. (51) reported no significant improvement in all cognitive tests when comparing IG with CG, whereas Tabka et al. found significant differences in both groups using MoCA. In addition, IG was found to be superior to the standard program alone in CG (39). One RCT compared the standard program as the CG and the combination of the standard program with CDE, finding that IG showed significant results within both groups in terms of utilizing MoCA and that the improvement was significant and favored IG (49).

#### 4.3.4 Effect of PR versus no control group on cognitive function

Two studies by Pereira et al. (53) and Bonnevie et al. (42) valuated the effect of the PR program without a compression group and identified post-PR improvements across various aspects. Pereira et al. found significant improvements in results for the Stroop Colour Word Test (*p* = 0.024) and the Rey Auditory Verbal Learning Test (RAVLT), which included three subtests: sum of trials one to five (*p* < 0.001), REY-IR (*p* = 0.005), and REY-DR (*p* = 0.022) (53). On the other hand, Bonnevie et al. found that participants without CI (initial MoCA > 26) showed no significant improvement post-PR. In contrast, participants with CI (initial MoCA < 26) showed significant post-PR improvements, especially in the memory component (*p* < 0.01) and in the final MoCA score (*p* < 0.01) (42). In addition, the total final MoCA scores of both groups showed significant improvement post-PR intervention (*p* < 0.01) (42).

### 4.4 Length of interventions employed in the studies

Descriptions of the intervention lengths for each study are provided in Table 1. These interventions lasted 3–12 weeks, except in one article, which extended its intervention to 6 months (51). One intervention lasted 3 weeks (54), another lasted 4 weeks (47), and another lasted 10 weeks (52). Two studies had interventions that were carried out over 6 weeks (40, 51); however, one of these studies extended the intervention duration to 6 months (51). Four studies conducted their interventions for 8 weeks (42, 48–50); however, in Lavoie et al.’s study (50), only the exercise component lasted 8 weeks. Three interventions were conducted over 12 weeks (39, 50, 53); in Lavoie et al.’s study (50), the self-management component lasted 12 weeks.

### 4.5 Measures of cognitive function among the studies

A total of 25 cognitive tests (or subtests) were used in the included studies, as shown in Table 2. Twenty-one tests specific to each domain and four general tests, including subtests representing multiple domains, were conducted. The 21 specific tests represented five cognitive domains. The cognitive tests employed by the included studies are shown in Table 3.

**Table 3:** Cognitive assessments of included studies.

Study	Cognitive test
Emery et al., 1998 (52)	Finger Tapping, Digit Vigilance, Digit Symbol, Trail Making Test Part A and Part B, and Verbal Fluency Test (Letter) (FAS).
Aquino et al., 2016 (47)	Verbal Fluency Test (Semantic), Attentive Matrices Test, RAVLT: Short delay recall (REY-IR) and Long delay recall (REY-DR), Drawing Copy Test I, Drawing Copy Test II, and Raven Test.
Incalzi et al., 2008 (51)	Digit Span (Visual span), Verbal Fluency Test (Letter) (FAS), Verbal span, Verbal competence (sentence construction), RAVLT: Short delay recall (REY-IR) and Long delay recall (REY-DR), Immediate visual memory, Drawing Copy Test I, Drawing Copy Test II, Raven Test, and MMSE test.
Lavoie et al., 2019 (50),	MoCA test.
Kaya et al., 2023 (49)	MoCA test.
Tabka et al.,2023 (39)	MoCA test.
Kozora et al., 2002 (54)	Digit Vigilance, Digit Span (Visual span), Digit Symbol, Trail Making Test Part B, Verbal Fluency Test (Letter) (FAS), Verbal Fluency Test (Semantic), Logical Memory (Story Retention), Visual Reproduction (Visual Retention), Paired Associates (Verbal Pairs), Clock Drawing, and Boston Naming Test
Pereira et al., 2011 (53)	Digit Span (Visual span), Stroop Colour Word Test, Verbal Fluency Test (Letter) (FAS), RAVLT: Sum of trials 1 to 5, Short delay recall (REY-IR), and Long delay recall (REY-DR)
Bonnevie et al., 2020 (42)	MoCA test.
France et al., 2021 (40)	MoCA test.
Cheng et al., 2022 (48)	MMSE test.

FAS, oral fluency by letters F, A, and S; RAVLT, Rey Auditory Verbal Learning Test; REY-IR, short delay recall; REY-DR, long delay recall; MoCA, Montreal Cognitive Assessment test; MMSE, the Mini Mental State Examination.

## 5 Discussion

This is the first systematic review to study the effects of PR on cognitive functions in patients with COPD. Among the 11 heterogeneous studies, PR programs resulted in significant improvements in cognitive function in nine studies, with significant improvements in almost all cognitive domains observed in five studies (39, 47, 49, 50, 53). Significant improvements in a single domain were noted in one study (52), and significant improvements among CI subjects only were found in three studies (40, 42, 54). However, PR did not improve cognitive domains in two studies (48, 51). The most frequent test used among the included studies was the MoCA test (39, 40, 42, 49, 50) and the most frequent component of the PR program was aerobic exercise (39, 40, 42, 47, 49–54).

PR programs significantly improved the following cognitive domains in COPD patients: visuospatial (39, 47, 49, 50), naming (39, 49, 50), attention (39, 47, 49, 50, 53), working memory (39, 53), language (39, 49, 50), intelligent and abstract reasoning (39, 47, 49, 50), learning and memory (39, 47, 49, 50, 53), and orientation (39, 49, 50). The improvements in cognitive functioning confirmed by several cognitive tests might be due to participation in a comprehensive PR program aligned with the American Association of Cardiovascular and Pulmonary Rehabilitation and the American Thoracic Society/European Respiratory Society guidelines (30, 55–57). In addition, some programs lasted 12 weeks (39, 50, 53) in accordance with international guidelines and recommendations (30, 55, 56). Etnier et al. revealed that the walking distance and cognitive capacity of COPD patients improved significantly following 12 weeks of exercise (58). Moreover, integrating a CogT program with a PR program might improve cognitive function. One study demonstrated the potential effectiveness of cognitive rehabilitation in improving cognitive function, QoL, and self-management ability in patients with COPD (59). Furthermore, integrating additional aerobic exercise programs, such as cycling, swimming, and dancing, with PR programs that include aerobic exercise is likely to improve cognitive abilities, as endurance exercises are well known as cornerstones of PR programs (30). Aerobic exercise has been shown to improve global cognitive function by possibly increasing brain volume and enhancing functional connectivity between the temporal cortices and a portion of the posterior frontal cortex (60). Emery et al. reported that executive function in COPD patients was the only cognitive domain to improve significantly after exercise training, and the authors believed this effect may be due to improved frontal lobe function, which would enable better executive functioning (52). The frontal lobe has previously been shown to improve cognitive function with exercise training (61). Significant improvements in cognitive domains among CI patients who underwent PR programs were identified in three studies (40, 42, 54). These cognitive domains include memory (42, 54), attention (54), and orientation (40). The practice effect for the cognitive test is proposed as the reason only CI subjects showed improvement, as the PR program lasted only 3 weeks, a period considered too short for pre- and post-test evaluation (54). In addition, the high risk of bias in the studies critically diverted the results toward the CI subjects, as this group was selected based on the most confounding factor: CI score (42).

Two studies noted no improvements in cognitive domains after the PR program (48, 51). This may be due to the use of a single PR component: respiratory muscle training (48). PR guidelines (30) recommend integrating multiple components rather than a single component, as this is beneficial for patients with COPD. The cornerstone of PR, exercise training, was not included in these two studies. Therefore, incorporating multiple components of the PR intervention may yield better results. Furthermore, Incalzi et al. stated that the elements of the CT program were not selected based on the participant’s defect, as the outcome of the baseline assessment revealed that the impairments were more involved with constructive functions than with orientation (51). Therefore, if the CT program were more focused on regaining or retaining constructive functions, it might result in improvements in cognitive function (51). Moreover, Winocur et al. found that subjects who entered CogTthree months after CI screening showed lower improvement than those who entered CogT immediately after screening (62). However, this was not the case in Incalzi et al.’s work, as the participants in this study began their training after the availability of a four-patient group, which suggests that some participants may have waited too long to start their post-screening training—this resulted in unequal starting points for post-screening training (51).

The specific elements of the program required to improve cognitive function in patients with COPD are not yet known, as the included studies varied in frequency and intensity, types of exercise, and program length. Our study results regarding the design of exercise programs align with a previous systematic review (43). The results of our study inform the proposal of a PR program based on the included studies, which show significant improvements in almost all cognitive domains among all COPD patients participating in the program. The suggested PR program elements include the following: PR duration must be four (47), eight (49, 50) or 12 weeks (39, 50, 53); intensity of exercise must range from moderate (39, 49) to high (47, 50) intensity; PR frequency should be set to three (39, 50, 53) to 5 days per week (47, 49); and the PR components aerobic exercise (39, 47, 49, 50, 53), resistance exercises (47, 50, 53), psychological sessions (53), education sessions (39, 53), breathing exercises (39, 47, 49), self-management (50), and balance exercises (47) must be included. These PR program elements were shown to be aligned with international PR recommendations (30, 55, 56).

The most frequently used cognitive test in the studies was the MoCA test, which was used in five studies (39, 40, 42, 49, 50), four of which (39, 40, 49, 50) reported their results as total scores and one of which (42) reported its results for each domain score separately. The studies justified their use of the MoCA test based on its efficacy, high sensitivity and specificity in the diagnosis, screening, and tracking of MCI (63). In 2014, the MoCA test was used for the first time to assess the cognitive function in COPD populations (64); therefore, only recent studies have used the test. According to previous studies, the MoCA test is superior to the MMSE test in detecting mild CI in COPD populations (65, 66); hence, this might explain why the two studies using the MMSE test did not achieve significant results (48, 51).

This systematic review has several strengths. It is the first systematic review to explore the effectiveness of PR programs in enhancing cognitive function in patients with COPD. All steps of the systematic review were conducted in accordance with the PRISMA guidelines. Moreover, this systematic review includes both RCTs and non-RCTs. To ensure rigorous methodological standards, the risk biases of the included studies were assessed using tools such as RoB 2 and ROBINS-I, as recommended by Cochrane. In addition, the review consists of all rehabilitation components of an intervention, not just exercises, as well as all types of neuropsychological battery tests.

This review has several limitations. The inclusion of only peer-reviewed studies published in English may have introduced both language and publication bias. Relevant studies published in other languages or reported in the grey literature may not have been identified, potentially leading to an overrepresentation. However, the findings were interpreted with caution.

In addition, one of the limitations in this review is the heterogeneity of the study’s design, the cognitive tests used, and the PR intervention elements examined, which make it challenging to conduct a meta-analysis. Although five studies assessed cognitive functions using the MoCA, a quantitative meta-analysis could not be conducted. This was primarily due to incomplete and inconsistent reporting of outcome data: only two studies provided the required summary statistics (mean and standard deviation) (49, 50), whereas the remaining three reported results were solely as *p*-values (39, 40, 42). Moreover, not all studies reported results for each MoCA domain separately; some presented MoCA scores as a total score for cognitive function (39, 49, 50). This makes it challenging to identify which domain has been improved by PR. Therefore, providing such details should help determine which domains have been improved and which have led to improvements in the total cognitive function score. In addition, these details will inform comparisons of MoCA results with those of other single neuropsychological batteries that assess the exact cognitive domains across different studies. Due to the heterogeneity of PR interventions comprising only a subset of traditional PR components (e.g., inspiratory muscle training alone) may exert different effects on cognitive outcomes compared with comprehensive, multidisciplinary PR programs, and that this should be considered when interpreting the findings and, in this study, the contribution to the cautious interpretation of the overall evidence was performed.

Future researchers should prioritize the thorough reporting of exercise intervention parameters, including the length of the program, the components used, the types of exercise employed in PR, the frequency and intensity of PR, and the duration of PR sessions. In addition, future studies should minimize the impact of practice on cognitive tests to enable practical findings to guide recommendations for PR programs used to improve cognitive function in COPD populations.

## 6 Conclusion

Patients with COPD are known to develop CI that progresses alongside the disease. PR programs constitute a vital non-pharmacological management method for COPD populations. PR is well known to improve cardiorespiratory fitness and QoL. However, its efficacy in improving cognitive function in COPD patients remains debated. This systematic review revealed that PR programs might improve cognitive function in patients with COPD, especially those also suffering from CI. However, the results of this study should be interpreted with caution, as the program design is heterogeneous and included PR interventions and cognitive tests. Future studies should unify the elements of PR interventions and neuropsychological batteries to enable a proper evaluation of the efficacy and application of PR programs.

## Data Availability

The original contributions presented in the study are included in the article/Supplementary material, further inquiries can be directed to the corresponding author.

## Glossary

AECOPD: Acute exacerbation of chronic obstructive pulmonary disease
AT: Aerobic training
CDE: Creative dance-based exercise training
CG: Control group
CI: Cognitive impairment
CogT: Cognitive training
COPD: Chronic obstructive pulmonary disease
CT: Combination of aerobic and strengthening training
EMT: Expiratory muscle training
FAS: Oral fluency test by letters F, A, and S
HR: Heart rate
HRQoL: Health-related quality of life
IG: Intervention group
IMT: Inspiratory muscle training
IS only: Impaired subject only
ITT: Intention-to-treat
MCI: Mild cognitive impairment
MIN: Minutes
MMSE: Mini-Mental State Examination
MoCA: Montreal cognitive assessment tool
NA: Not available
NS: Not significant
PR: Pulmonary rehabilitation
PRISMA 2020: Preferred Reporting Items for Systematic Reviews and Meta-Analysis 2020
PT: Physiotherapy
QoL: Quality of life
RAVLT: Rey Auditory Verbal Learning Test
RCT: Randomized control trials
REY-DR: Long delay recall
REY-IR: Short delay recall
ROB 2: Risk of Bias in Randomized Trials
ROBIN-I: Risk of Bias In Non-Randomized Studies - of Interventions
SMBM: Self-Management Behavior Modification
SMBM+P: Self-Management Behavior Modification with Placebo
SMBM+T: Self-Management Behavior Modification and Tiotropium 5 μg
SMBM+T/O: Self-Management Behavior Modification with Tiotropium/Olodaterol 5/5 μg
SBMB+T/O+ExT: Self-Management Behavior Modification with Tiotropium/Olodaterol 5/5 μg and exercise training
W: Watt
WAIS-R: Wechsler Adult Intelligence Scale – Revised
WMS-R: Wechsler Memory Scale – Revised
1-RM: One Repetition Maximum

## References

[1] Lezak MD. Neuropsychological Assessment. New York: Oxford University Press (2004).

[2] Cleutjens FA Janssen DJ Ponds RW Dijkstra JB Wouters EF. COgnitive-pulmonary disease. Biomed Res Int. (2014) 2014:697825. doi: 10.1155/2014/697825, 24738069 PMC3971492

[3] Andrianopoulos V Gloeckl R Vogiatzis I Kenn K. Cognitive impairment in COPD: should cognitive evaluation be part of respiratory assessment? Breathe (Sheff). (2017) 13:e1–9. doi: 10.1183/20734735.001417, 29184593 PMC5702891

[4] Gottfredson LS. Why g matters: the complexity of everyday life. Intelligence. (1997) 24:79–132. doi: 10.1016/s0160-2896(97)90014-3

[5] Sachdev PS Blacker D Blazer DG Ganguli M Jeste DV Paulsen JS . Classifying neurocognitive disorders: the DSM-5 approach. Nat Rev Neurol. (2014) 10:634–42. doi: 10.1038/nrneurol.2014.181, 25266297

[6] Siraj RA. Comorbid cognitive impairment in chronic obstructive pulmonary disease (COPD): current understanding, risk factors, implications for clinical practice, and suggested interventions. Medicina (Kaunas). (2023) 59:732. doi: 10.3390/medicina59040732, 37109690 PMC10146750

[7] Dhakal A Bobrin BD. "Cognitive deficits". In: StatPearls. Treasure Island (FL): StatPearls Publishing LLC (2023) 32644478

[8] Venkatesan P. GOLD COPD report: 2023 update. Lancet Respir Med. (2023) 11:18. doi: 10.1016/S2213-2600(22)00494-5, 36462509

[9] WHO. (2023). World Health Organization: Chronic obstructive pulmonary disease (COPD). Available online at: https://www.who.int/news-room/fact-sheets/detail/chronic-obstructive-pulmonary-disease-(copd) (Accessed November 16, 2023).

[10] Huber MB Wacker ME Vogelmeier CF Leidl R. Comorbid influences on generic health-related quality of life in COPD: a systematic review. PLoS One. (2015) 10:e0132670. doi: 10.1371/journal.pone.0132670, 26168154 PMC4500578

[11] Santos NC Miravitlles M Camelier AA Almeida VDC Maciel RRBT Camelier FWR. Prevalence and impact of comorbidities in individuals with chronic obstructive pulmonary disease: a systematic review. Tuberculosis Respir Dis. (2022) 85:205–20. doi: 10.4046/trd.2021.0179, 35618259 PMC9263346

[12] Vanfleteren LE Spruit MA Groenen M Gaffron S van Empel VP Bruijnzeel PL . Clusters of comorbidities based on validated objective measurements and systemic inflammation in patients with chronic obstructive pulmonary disease. Am J Respir Crit Care Med. (2013) 187:728–35. doi: 10.1164/rccm.201209-1665OC, 23392440

[13] Torres-Sánchez I Rodríguez-Alzueta E Cabrera-Martos I López-Torres I Moreno-Ramírez MP Valenza MC. Cognitive impairment in COPD: a systematic review. J Bras Pneumol. (2015) 41:182–90. doi: 10.1590/S1806-37132015000004424, 25909154 PMC4428856

[14] Lindbergh CA Dishman RK Miller LS. Functional disability in mild cognitive impairment: a systematic review and Meta-analysis. Neuropsychol Rev. (2016) 26:129–59. doi: 10.1007/s11065-016-9321-5, 27393566

[15] Yohannes AM Chen W Moga AM Leroi I Connolly MJ. Cognitive impairment in chronic obstructive pulmonary disease and chronic heart failure: a systematic review and Meta-analysis of observational studies. J Am Med Dir Assoc. (2017) 18:451.e1–451.e11. doi: 10.1016/j.jamda.2017.01.014 28292570

[16] van Beers M Janssen DJA Gosker HR Schols A. Cognitive impairment in chronic obstructive pulmonary disease: disease burden, determinants and possible future interventions. Expert Rev Respir Med. (2018) 12:1061–74. doi: 10.1080/17476348.2018.1533405, 30296384

[17] Wang M Wang Y Wang Z Ren Q. The abnormal alternations of brain imaging in patients with chronic obstructive pulmonary disease: a systematic review. J Alzheimers Dis Rep. (2023) 7:901–19. doi: 10.3233/ADR-220083, 37662615 PMC10473125

[18] Rasch B Born J. About sleep's role in memory. Physiol Rev. (2013) 93:681–766. doi: 10.1152/physrev.00032.2012, 23589831 PMC3768102

[19] Eide PK Vinje V Pripp AH Mardal KA Ringstad G. Sleep deprivation impairs molecular clearance from the human brain. Brain. (2021) 144:863–74. doi: 10.1093/brain/awaa443, 33829232

[20] Olaithe M Bucks RS Hillman DR Eastwood PR. Cognitive deficits in obstructive sleep apnea: insights from a meta-review and comparison with deficits observed in COPD, insomnia, and sleep deprivation. Sleep Med Rev. (2018) 38:39–49. doi: 10.1016/j.smrv.2017.03.005, 28760549

[21] Pelgrim CE Peterson JD Gosker HR Schols A van Helvoort A Garssen J . Psychological co-morbidities in COPD: targeting systemic inflammation, a benefit for both? Eur J Pharmacol. (2019) 842:99–110. doi: 10.1016/j.ejphar.2018.10.001, 30336140

[22] Majid H Nadeem T. "Anxiety and depression in COPD patients". In: Sharafkhaneh A Yohannes AM Hanania NA Kunik ME, editors. Depression and Anxiety in Patients with Chronic Respiratory Diseases. New York, NY: Springer New York (2017). p. 57–72.

[23] Kakkera K Padala KP Kodali M Padala PR. Association of chronic obstructive pulmonary disease with mild cognitive impairment and dementia. Curr Opin Pulm Med. (2018) 24:173–8. doi: 10.1097/MCP.0000000000000458, 29232279

[24] Hoiland RL Mladinov S Barak OF Willie CK Mijacika T Stembridge M . Oxygen therapy improves cerebral oxygen delivery and neurovascular function in hypoxaemic chronic obstructive pulmonary disease patients. Exp Physiol. (2018) 103:1170–7. doi: 10.1113/EP086994, 29978513

[25] Thakur N Blanc PD Julian LJ Yelin EH Katz PP Sidney S . COPD and cognitive impairment: the role of hypoxemia and oxygen therapy. Int J Chron Obstruct Pulmon Dis. (2010) 5:263–9. doi: 10.2147/copd.s10684 20856825 PMC2939681

[26] Areza-Fegyveres R Kairalla RA Carvalho CRR Nitrini R. Cognition and chronic hypoxia in pulmonary diseases. Dement Neuropsychol. (2010) 4:14–22. doi: 10.1590/S1980-57642010DN40100003, 29213655 PMC5619525

[27] Baird C Lovell J Johnson M Shiell K Ibrahim JE. The impact of cognitive impairment on self-management in chronic obstructive pulmonary disease: a systematic review. Respir Med. (2017) 129:130–9. doi: 10.1016/j.rmed.2017.06.006, 28732820

[28] Alison JA McKeough ZJ Johnston K McNamara RJ Spencer LM Jenkins SC . Australian and New Zealand pulmonary rehabilitation guidelines. Respirology. (2017) 22:800–19. doi: 10.1111/resp.13025, 28339144

[29] Rochester CL Vogiatzis I Holland AE Lareau SC Marciniuk DD Puhan MA . An official American Thoracic Society/European Respiratory Society policy statement: enhancing implementation, use, and delivery of pulmonary rehabilitation. Am J Respir Crit Care Med. (2015) 192:1373–86. doi: 10.1164/rccm.201510-1966ST, 26623686

[30] Spruit MA Singh SJ Garvey C ZuWallack R Nici L Rochester C . An official American Thoracic Society/European Respiratory Society statement: key concepts and advances in pulmonary rehabilitation. Am J Respir Crit Care Med. (2013) 188:e13–64. doi: 10.1164/rccm.201309-1634ST, 24127811

[31] Bolton CE Bevan-Smith EF Blakey JD Crowe P Elkin SL Garrod R . British Thoracic Society guideline on pulmonary rehabilitation in adults. Thorax. (2013) 68:ii1–ii30. doi: 10.1136/thoraxjnl-2013-203808 23880483

[32] McCarthy B Casey D Devane D Murphy K Murphy E Lacasse Y. Pulmonary rehabilitation for chronic obstructive pulmonary disease. Cochrane Database Syst Rev. (2015) 2015:Cd003793. doi: 10.1002/14651858.CD003793.pub3 25705944 PMC10008021

[33] Li W Pu Y Meng A Zhi X Xu G. Effectiveness of pulmonary rehabilitation in elderly patients with COPD: a systematic review and meta-analysis of randomized controlled trials. Int J Nurs Pract. (2019) 25:e12745. doi: 10.1111/ijn.12745, 31268214

[34] Yang J Lin R Xu Z Zhang H. Significance of pulmonary rehabilitation in improving quality of life for subjects with COPD. Respir Care. (2019) 64:99–107. doi: 10.4187/respcare.06353, 30578361

[35] Higashimoto Y Ando M Sano A Saeki S Nishikawa Y Fukuda K . Effect of pulmonary rehabilitation programs including lower limb endurance training on dyspnea in stable COPD: a systematic review and meta-analysis. Respir Investig. (2020) 58:355–66. doi: 10.1016/j.resinv.2020.05.010, 32660900

[36] Hindelang M Kirsch F Leidl R. Effectiveness of non-pharmacological COPD management on health-related quality of life - a systematic review. Expert Rev Pharmacoecon Outcomes Res. (2020) 20:79–91. doi: 10.1080/14737167.2020.1734455, 32098530

[37] Corhay JL Dang DN Van Cauwenberge H Louis R. Pulmonary rehabilitation and COPD: providing patients a good environment for optimizing therapy. Int J Chron Obstruct Pulmon Dis. (2014) 9:27–39. doi: 10.2147/COPD.S52012, 24368884 PMC3869834

[38] Ryrsø CK Godtfredsen NS Kofod LM Lavesen M Mogensen L Tobberup R . Lower mortality after early supervised pulmonary rehabilitation following COPD-exacerbations: a systematic review and meta-analysis. BMC Pulm Med. (2018) 18:154. doi: 10.1186/s12890-018-0718-1, 30219047 PMC6139159

[39] Tabka O Sanaa I Mekki M Acheche A Paillard T Trabelsi Y. Effect of a pulmonary rehabilitation program combined with cognitive training on exercise tolerance and cognitive functions among Tunisian male patients with chronic obstructive pulmonary disease: a randomized controlled trial. Chron Respir Dis. (2023) 20:14799731231201643. doi: 10.1177/14799731231201643, 37691169 PMC10494516

[40] France G Orme MW Greening NJ Steiner MC Chaplin EJ Clinch L . Cognitive function following pulmonary rehabilitation and post-discharge recovery from exacerbation in people with COPD. Respir Med. (2021) 176:106249. doi: 10.1016/j.rmed.2020.106249, 33253973

[41] Andrianopoulos V Gloeckl R Schneeberger T Jarosch I Vogiatzis I Hume E . Benefits of pulmonary rehabilitation in COPD patients with mild cognitive impairment - a pilot study. Respir Med. (2021) 185:106478. doi: 10.1016/j.rmed.2021.106478, 34038843

[42] Bonnevie T Medrinal C Combret Y Debeaumont D Lamia B Muir JF . Mid-term effects of pulmonary rehabilitation on cognitive function in people with severe chronic obstructive pulmonary disease. Int J Chron Obstruct Pulmon Dis. (2020) 15:1111–21. doi: 10.2147/COPD.S249409, 32546999 PMC7245438

[43] Desveaux L Harrison SL Gagnon JF Goldstein RS Brooks D Pepin V. Effects of exercise training on cognition in chronic obstructive pulmonary disease: a systematic review. Respir Med. (2018) 139:110–6. doi: 10.1016/j.rmed.2018.05.006, 29857994

[44] Page MJ McKenzie JE Bossuyt PM Boutron I Hoffmann TC Mulrow CD . The PRISMA 2020 statement: an updated guideline for reporting systematic reviews. BMJ. (2021) 372:n71. doi: 10.1136/bmj.n71, 33782057 PMC8005924

[45] Sterne JAC Savović J Page MJ Elbers RG Blencowe NS Boutron I . RoB 2: a revised tool for assessing risk of bias in randomised trials. BMJ. (2019) 366:l4898. doi: 10.1136/bmj.l4898, 31462531

[46] Sterne JA Hernán MA Reeves BC Savović J Berkman ND Viswanathan M . Robins-i: a tool for assessing risk of bias in non-randomised studies of interventions. BMJ. (2016) 355:i4919. doi: 10.1136/bmj.i4919 27733354 PMC5062054

[47] Aquino G Iuliano E di Cagno A Vardaro A Fiorilli G Moffa S . Effects of combined training vs aerobic training on cognitive functions in COPD: a randomized controlled trial. Int J Chron Obstruct Pulmon Dis. (2016) 11:711–8. doi: 10.2147/COPD.S96663, 27110107 PMC4831596

[48] Cheng YY Lin SY Hsu CY Fu PK. Respiratory muscle training can improve cognition, lung function, and diaphragmatic thickness fraction in male and non-obese patients with chronic obstructive pulmonary disease: a prospective study. J Pers Med. (2022) 12:475. doi: 10.3390/jpm12030475 35330474 PMC8955729

[49] Kaya M Gurses HN Ucgun H Okyaltirik F. Effects of creative dance on functional capacity, pulmonary function, balance, and cognition in COPD patients: a randomized controlled trial. Heart Lung. (2023) 58:13–20. doi: 10.1016/j.hrtlng.2022.10.017, 36335909

[50] Lavoie KL Sedeno M Hamilton A Li PZ De Sousa D Troosters T . Behavioural interventions targeting physical activity improve psychocognitive outcomes in COPD. ERJ Open Res. (2019) 5:00013. doi: 10.1183/23120541.00013-2019, 31720294 PMC6826247

[51] Incalzi RA Corsonello A Trojano L Pedone C Acanfora D Spada A . Cognitive training is ineffective in hypoxemic COPD: a six-month randomized controlled trial. Rejuvenation Res. (2008) 11:239–50. doi: 10.1089/rej.2007.0607, 18279034

[52] Emery CF Schein RL Hauck ER MacIntyre NR. Psychological and cognitive outcomes of a randomized trial of exercise among patients with chronic obstructive pulmonary disease. Health Psychol. (1998) 17:232–40. 9619472 10.1037//0278-6133.17.3.232

[53] Pereira ED Viana CS Taunay TC Sales PU Lima JW Holanda MA. Improvement of cognitive function after a three-month pulmonary rehabilitation program for COPD patients. Lung. (2011) 189:279–85. doi: 10.1007/s00408-011-9303-6, 21656143

[54] Kozora E Tran ZV Make B. Neurobehavioral improvement after brief rehabilitation in patients with chronic obstructive pulmonary disease. J Cardpulm Rehabil. (2002) 22:426–30. doi: 10.1097/00008483-200211000-00008 12464831

[55] AACVPR AAoCaPR. Guidelines for Pulmonary Rehabilitation Programs-5th Edition AACVPR, American Association of Cardiovascular and Pulmonary Rehabilitation. Champaign, IL, USA: Human Kinetics, Inc. (2013).

[56] Nici L Donner C Wouters E Zuwallack R Ambrosino N Bourbeau J . American Thoracic Society/European Respiratory Society statement on pulmonary rehabilitation. Am J Respir Crit Care Med. (2006) 173:1390–413. doi: 10.1164/rccm.200508-1211ST 16760357

[57] Shenoy M. A. Paul V. (2023) Pulmonary Rehabilitation: National Library of Medicine, StatPearls 2023. Available online at: https://www.ncbi.nlm.nih.gov/books/NBK563166/

[58] Etnier JL Berry M. Fluid intelligence in an older COPD sample after short- or long-term exercise. Med Sci Sports Exerc. (2001) 33:1620–8. doi: 10.1097/00005768-200110000-00002 11581543

[59] Park MO Oh HS Seo WS. Effects of a cognitive rehabilitation programme on cognitive function, self-management and quality of life in patients with chronic obstructive pulmonary disease. Int J Nurs Pract. (2021) 27:e12932. doi: 10.1111/ijn.12932, 33830593

[60] Suzuki T Shimada H Makizako H Doi T Yoshida D Ito K . A randomized controlled trial of multicomponent exercise in older adults with mild cognitive impairment. PLoS One. (2013) 8:e61483. doi: 10.1371/journal.pone.0061483, 23585901 PMC3621765

[61] Shin M-K. Effects of an exercise program on frontal lobe cognitive function in elders. J Korean Acad Nurs. (2009) 39:107–15. doi: 10.4040/jkan.2009.39.1.107, 19265317

[62] Winocur G Craik FI Levine B Robertson IH Binns MA Alexander M . Cognitive rehabilitation in the elderly: overview and future directions. J Int Neuropsychol Soc. (2007) 13:166–71. doi: 10.1017/S1355617707070191 17166315

[63] Smith T Gildeh N Holmes C. The Montreal cognitive assessment: validity and utility in a memory clinic setting. Can J Psychiatr. (2007) 52:329–32. doi: 10.1177/070674370705200508, 17542384

[64] Crişan AF Oancea C Timar B Fira-Mladinescu O Crişan A Tudorache V. Cognitive impairment in chronic obstructive pulmonary disease. PLoS One. (2014) 9:e102468. doi: 10.1371/journal.pone.0102468, 25033379 PMC4102489

[65] Villeneuve S Pepin V Rahayel S Bertrand J-A de Lorimier M Rizk A . Mild cognitive impairment in moderate to severe COPD: a preliminary study. Chest. (2012) 142:1516–23. doi: 10.1378/chest.11-3035, 23364388

[66] Nasreddine ZS Phillips NA Bédirian V Charbonneau S Whitehead V Collin I . The Montreal cognitive assessment, MoCA: a brief screening tool for mild cognitive impairment. J Am Geriatr Soc. (2005) 53:695–9. doi: 10.1111/j.1532-5415.2005.53221.x, 15817019

